# Occurrence and seasonal dynamics of RNA viral genotypes in three contrasting temperate lakes

**DOI:** 10.1371/journal.pone.0194419

**Published:** 2018-03-15

**Authors:** Ian Hewson, Kalia S. I. Bistolas, Jason B. Button, Elliot W. Jackson

**Affiliations:** Department of Microbiology, Cornell University, Ithaca, NY United States of America; Oklahoma State University, UNITED STATES

## Abstract

Decades of research have demonstrated the crucial importance of viruses in freshwater ecosystems. However, few studies have focused on the seasonal dynamics and potential hosts of RNA viruses. We surveyed microbial-sized (i.e. 5–0.2 μm) mixed community plankton transcriptomes for RNA viral genomes and investigated their distribution between microbial and macrobial plankton over a seasonal cycle across three temperate lakes by quantitative reverse transcriptase PCR (qRT-PCR). A total of 30 contigs bearing similarity to RNA viral genomes were recovered from a global assembly of 30 plankton RNA libraries. Of these, only 13 were found in >2 libraries and recruited >100 reads (of 9.13 x 10^7^ total reads), representing several picornaviruses, two tobamoviruses and a reovirus. We quantified the abundance of four picornaviruses and the reovirus monthly from August 2014 to May 2015. Patterns of viral abundance in the >5 μm size fraction and representation in microbial-sized community RNA libraries over time suggest that one picornavirus genotype (TS24835) and the reovirus (TS148892) may infect small (<5 μm) eukaryotic microorganisms, while two other picornaviruses (TS24641 and TS4340) may infect larger (>5 μm) eukaryotic microorganisms or metazoa. Our data also suggest that picornavirus TS152062 may originate from an allochthonous host. All five viral genotypes were present in at least one size fraction across all 3 lakes during the year, suggesting that RNA viruses may easily disperse between adjacent aquatic habitats. Our data therefore demonstrate that RNA viruses are widespread in temperate lacustrine ecosystems, and may provide evidence of viral infection in larger eukaryotes (including metazoa) inhabiting the lakes.

## Introduction

Nearly 30 years of research has highlighted the crucial role of viruses in aquatic ecosystems, where they cause significant mortality of microbial and metazoan hosts, facilitate gene exchange, and influence elemental cycling [1–5]. Viral communities in aquatic ecosystems are enormously diverse, comprising genotypes that infect bacteria, eukaryotic microorganisms, and multicellular organisms [6–13]. While aquatic viruses may bear either DNA or RNA genomes, most studies to date have focused on identification and characterization of DNA viruses [14–20]. Understanding of RNA viral diversity has undergone considerable revision in the last 5 years with the application of shotgun-based sequencing (i.e. metagenomics) approaches to size-fractioned environmental samples [21–27] and whole tissues of non-model metazoa [28–32]. These studies have revealed a wide array of viruses that expand known taxonomy of RNA viruses [28]. Despite a growing appreciation for the potential significance of RNA viruses to aquatic food web processes and host biodiversity, there remains little information about the taxonomic and genetic composition, biogeography and seasonal recurrence of RNA viruses across many aquatic habitats.

Temperate lacustrine ecosystems represent interesting habitats in which to study the diversity and temporal dynamics of aquatic viruses for several reasons. Lakes receive constant and episodic allochthonous inputs from terrestrial environments (i.e. runoff). Lakes at high latitudes experience distinct seasonal shifts in biological phenomena, including seasonal (primarily spring) blooms, post-bloom clear-water phases, and ice cover in winter. They are also subject to greater variability in natural disturbance events (e.g. ice cover, storms, seiches, etc.) than lakes at lower latitudes. Lakes also experience various mixing regimes, from meromictic to polymictic which influence their productivity and microbial ecology. These distinct mixing regimes and productivity cycles provide ideal conditions to examine dynamics of viral groups by providing stark temporal changes in host abundance.

The composition of viral communities in aquatic ecosystems is a function of production of new virus particles through autochthonous infection and import from allochthonous sources and their decay [33–38]. Runoff delivers viruses from surrounding non-aquatic habitats to lake waters. Lakes also receive inputs from airborne animals which facilitate dispersal of other organisms and presumably their associated microbiota and viruses [39]. Decay of aquatic viruses is mostly attributed to labile organic matter which presumably includes nucleases and proteases [40], and sunlight which degrades capsids and genomes [41]. Viral particles may also adsorb to surfaces of particles and sink into deeper waters and sediments [42]. Most studies of marine viral decay converge on a rapid 2–4% h^-1^ [1, 43], while in freshwater ecosystems study of individual genotypes indicated slower decay rates (0.1–1.5% h^-1^) [33, 44], and some types of viruses (e.g. nucleopolyhedroviruses) may persist for months-years without significant decay [45, 46]. Thus, the assemblage of viruses present in lake waters may represent viruses infecting autochthonous hosts, those infecting allochthonous hosts that are recently arrived, and viruses that accumulate in the lake and are resistant to decay.

Numerous studies in the last two decades have focused on surveying viral composition in aquatic ecosystems to understand patterns of diversity as they relate to environmental parameters and hosts [14–27]. Most studies to date have approached viral diversity via shotgun sequencing of mixed-community viral genomes (i.e. viral metagenomics), where viruses are purified from tissues or directly from environmental samples, their nucleic acids extracted, and then sequenced [47, 48]. Viral metagenomics was first applied to marine plankton [13] and human fecal samples [49]. Since the advent of economical third-generation sequencing, the approach has become common in studies of viral diversity worldwide. RNA viral diversity in freshwater habitats was first determined using metagenomics in 2009 [50], and has since been applied elsewhere [51–54]. Early study of community transcriptomics from environmental samples highlighted the unexpectedly large proportion of sequence reads associated with viruses [55]. More recently, shotgun sequencing of invertebrate transcriptomes (‘RNAseq’) has confirmed that transcript-based sequencing efforts are an effective means for retrieving viral genomes [29, 56].

The purpose of this study was to understand RNA viral composition in three temperate lakes with variable hydrology and allochthonous inputs. We first surveyed RNA viral genomes using a community transcriptomics approach targeting microbial-sized (0.2–5 μm) material. We identified RNA viral genomes within transcriptomes, then evaluated their seasonal distribution in the lakes on particles >5 μm (i.e. present in aggregates or particles, large microbial eukaryotes or metazoa). Our results revealed that several RNA viral genotypes are temporally transient and lake-specific, may relate to precipitation and runoff, and may follow seasonal patterns of productivity.

## Methods

### Description of Finger Lakes

The Finger Lakes (New York State, USA) are a series of 11 freshwater lakes oriented approximately north-south, ranging in length from 4.8 km to 64 km, and in maximum depth from 29 m to 188 m. Cayuga Lake, which is the longest of the Finger Lakes, is mesotrophic [57], having received inputs of fertilizer and runoff from agricultural lands in the region [58]. Seneca Lake, which is the second largest and deepest of the Finger Lakes, is also mesotrophic [59]. Owasco Lake, which is considerably smaller (18km long and 54m deep), has a large catchment (540 km^2^) relative to its size, and is surrounded by agricultural land [59]. As a consequence, it is more eutrophic than other lakes in the region [59].

### Sample collection

Samples were collected monthly between September 2014 and August 2015 from three locations: Owasco Lake (42.754468^o^N, 76.470780^o^W; OL); Seneca Lake (42.618192^o^N, 76.878926^o^W; SL) and Cayuga Lake (42.537017^o^N, 76.550545^o^W; CL) (S1 Table). In February 2015 lake water was inaccessible due to ice. Water was collected from a pier at each location in approximately 1 m water using a sample-rinsed bucket and placed into duplicate sample-rinsed 20 L HDPE cubitainers. Temperature, conductivity and O_2_ were measured during each sampling expedition using a hand-held YSI probe. Samples were transported in coolers to maintain ambient water temperature to the lab at Cornell University. Water samples were subsampled within 2 h collection for bacterial abundance (~10 mL), chlorophyll a concentration (~ 5 L), and plankton RNA (~20 L). No specific permissions were required for these locations/activities, and this work did not involve endangered or protected species.

### Chlorophyll *a* analyses

Duplicate samples for chlorophyll *a* analysis (0.1–1 L) were diafiltered through 47 mm diameter GF/F filters, which were subsequently encased in alumninium foil and frozen at -20°C prior to analysis. Samples for chlorophyll *a* were analysed by the acetone extraction-fluorometry approach [60]. Filters were immersed in 10 mL of 90% acetone in 13x100mm borosilicate glass tubes and incubated overnight in darkness at -20°C. Filters were subsequently removed and the chlorophyll *a* concentration determined without correction for phaeophytin in a hand-held Turner Designs Fluorometer.

### Bacterioplankton abundance

Samples for bacterioplankton abundance (10 mL) were collected and fixed in 2% formalin and stored at 4°C prior to analysis. Preparation of slides for bacterial enumeration occurred 7–14 d after sample collection. Bacterial abundance was determined in fixed samples by DAPI epifluorescence microscopy [61]. Duplicate subsamples (1 mL) of water were stained in darkness with DAPI (20 μL mL [lake water]^-1^) for 2 min before being filtered through black 0.2 μm polycarbonate filters. The filters were then mounted on glass slides using PBS:Glycerol (1:1) as mountant. Slides were kept frozen prior to microscopy. Bacteria were counted on an Olympus BX-51 epifluorescence microscope under blue light excitation and at 1000X magnification. Ten random fields on each slide containing >200 cells were counted for each slide and abundance calculated from the average bacterial abundance per field.

### Preparation of plankton RNA

Duplicate samples for plankton RNA (0.5–2 L, depending on sampling date; S1 Table) were serially filtered through 142 mm diameter 5 μm (Isopore) and 0.2 μm (Durapore) filters using positive air pressure. The filters were immediately frozen at -80°C prior to downstream analyses. Plankton RNA libraries were prepared from the 0.2 μm size fraction filters following the protocols of Hewson et al. [62]. RNA was extracted from the 142 mm filters in ZR RNA Buffer (Zymo Research) and subject to bead beating for 2 min. The homogenate was processed through the ZR RNA Mini Isolation Kit. After extraction, RNA was depleted of contaminating DNA using the DNA-free RNA Kit (Zymo Research), and treated with terminator exonuclease (Epicentre) to increase the ratio of mRNA relative to rRNA. Following treatment, samples were amplified using the TransPlex kit (Sigma Aldrich). Plankton RNA libraries were sequenced at the Cornell Biotechnology Resource Center following library preparation (Nextera). Each plankton RNA library was run on 1/16 of an Illumina MiSeq paired-end 250nt lane.

### Bioinformatic analyses of environmental RNA libraries

Sequence libraries were first vetted for poor quality sequence (Ambiguous nucleotides ≤1), trimmed for adapters and length using the CLC Genomics Workbech 4.0. Following this, libraries were assembled using the program’s *de novo* assembly algorithm with default settings and with contig lengths ≥ 500 nt, minimum overlap 0.2 and minimum similarity 0.8. To identify contigs which may represent RNA virus genomes or genome fragments, all contigs were first subject to tBLASTx against an in-house database of complete RNA genomes collected from NCBI GenBank (keyword search “RNA Virus” with filter “complete genome” as of May 2016). Contigs matching the RNA viral database at an e-value < 10^−10^ were then subject to BLASTn against the non-redundant (*nr*) database at NCBI to confirm viral identity. RNA viral contigs (i.e. those matching RNA genomes via tBLASTx) were then subject to fragment recruitment using a minimum overlap of 0.1 and minimum similarity of 0.95 for all environmental RNA libraries. Only contigs recruiting ≥ 100 reads across ≥ 2 libraries were considered further. Contigs fulfilling these criteria were then subject to tBLASTx against the *nr* database to identify their phylogenetically closest relative. Sequence data is available at NCBI under BioProject accession PRJNA417956.

### Determination of viral genotype abundance by qRT-PCR

Five candidate RNA viral phylotypes were selected for focus that were represented in ≥ 5 libraries (Table 1; NCBI accessions MG550036—MG550040). The abundance of RNA viral genotypes was determined by quantitative reverse transcriptase PCR (qRT-PCR). Total RNA was extracted from 5 μm filters using the Zymo RNA Mini Isolation Kit. RNA was then depleted of contaminating DNA by the Zymo DNA-free RNA kit, and subsequently converted to cDNA using Superscript III reverse transcriptase. Primers and probes were designed based on capsid proteins for the 5 genotypes using the program Primer3 (http://www.primer3.com) [63]. qRT-PCR was performed in duplicate reactions where each reaction contained 1X SSO Probes Universal Master Mix (Bio-Rad), 200pmol of each primer (S2 Table) and 5’-FAM labeled probe (3’-TAMRA as quencher), and 2 μl of template cDNA in 25μl reactions. Each run was performed with 4 no template controls and duplicate oligonucleotide standards over 8 orders of magnitude copy number. Quantity was determined by comparison of cycle threshold values against the standards and multiplied by 2 to account for single-stranded standards. The practical detection threshold of all 5 primer/probe pairs was 1 genotype copies reaction^-1^ based on linearity of standards and absence of amplification in no template controls; this equates to <0.001 genotype copies mL^-1^ based on the volumes of water filtered.

**Table 1:** Contigs matching known viruses by BLASTx against the non-redundant (*nr*) database at NCBI. The closest cultured virus and uncultured virus relatives are indicated.

Contig ID	Contig Length (nt)	Closest Cultured Virus	Genbank Accession	E-Value	Closest Uncultured Virus	Genbank Accession	E-Value
TS152062*	416	*Spodoptera exigua* virus AKJ-2014	AHX00963	3.00E-17	Wenling crustacean virus 6	YP_009336650.1	3.00E-31
TS189778	801	*Asterionellopsis glacialis* RNA virus	YP_009047194.1	2.00E-08	Picornavirales Q_sR_OV_019	ASG92548.1	4.00E-22
TS24641*	5860	*Delisea pulchra* RNA virus	YP_009227212.1	3.00E-90	Beihai paphia shell virus 2	YP_009333342.1	9.00E-157
TS148892*	997	Orungo virus	AFX73387.1	5.00E-17	-	-	-
TS117426	862	*Chara australis* virus	AEJ33768.1	7.00E-81	-	-	-
TS147687	650	*Asterionellopsis glacialis* RNA virus	YP_009047194.1	2.00E-27	Beihai picorna-like virus 14	YP_009333555.1	3.00E-30
TS24757	4491	Bat dicibavirus	YP_009345907.1	2.00E-114	Sanxia picorna-like virus 5	YP_009337800.1	3.00E-156
TS24835*	7983	Bat dicibavirus	YP_009345907.1	1.00E-78	Beihai picorna-like virus 47	YP_009333390.1	0.00E+00
TS143854	582	Bat dicibavirus	YP_009345907.1	1.00E-38	Sanxia picorna-like virus 5	YP_009337800.1	3.00E-44
TS139783	540	Bat dicibavirus	YP_009345907.1	5.00E-07	Sanxia picorna-like virus 5	YP_009337800.1	1.00E-14
TS4340*	7570	Slow bee paralysis virus	YP_003622540.1	6.00E-76	Hubei picorna-like virus 26	YP_009337003.1	7.00E-75
TS145986	587	*Chara australis* virus	AEJ33768.1	2.00E-79	-	-	-
TS132784	649	*Chaetoceros tenuissimus* RNA virus type-II	YP_009111337.1	1.00E-12	Beihai picorna-like virus 29	APG77924.1	2.00E-20

The five contigs chosen for qPCR analysis are indicated by *.

### Statistical analyses

All statistical analyses were performed in Microsoft Excel using the XLStat plugin (Addinsoft SARL). Pairwise correlation significance (Pearsons ρ) α values were corrected for Type II error by dividing 0.05 by the number of comparisons made.

## Results and discussion

RNA viral communities inhabiting lacustrine ecosystems are a milieu of temporally and spatially variable agents which may arise from both aquatic infections and from infection of hosts in non-aquatic habitats. Our data provide evidence that RNA viruses observed in microbial-sized (0.2–5 μm) plankton RNA libraries may, in fact, represent viruses of larger (>5 μm) microbial eukaryotes, aggregates of eukaryotes, or metazoa. Some RNA viral genotypes had a seasonal trend which may be related to overall patterns of lake productivity (i.e. fall/spring phytoplankton blooms) and precipitation/runoff. Our data provide evidence that several RNA viral genotypes were dispersed between all three lakes, but their presence in different size fractions varied between lakes.

### Microbial biomass and physicochemical conditions

**Fig 1:**
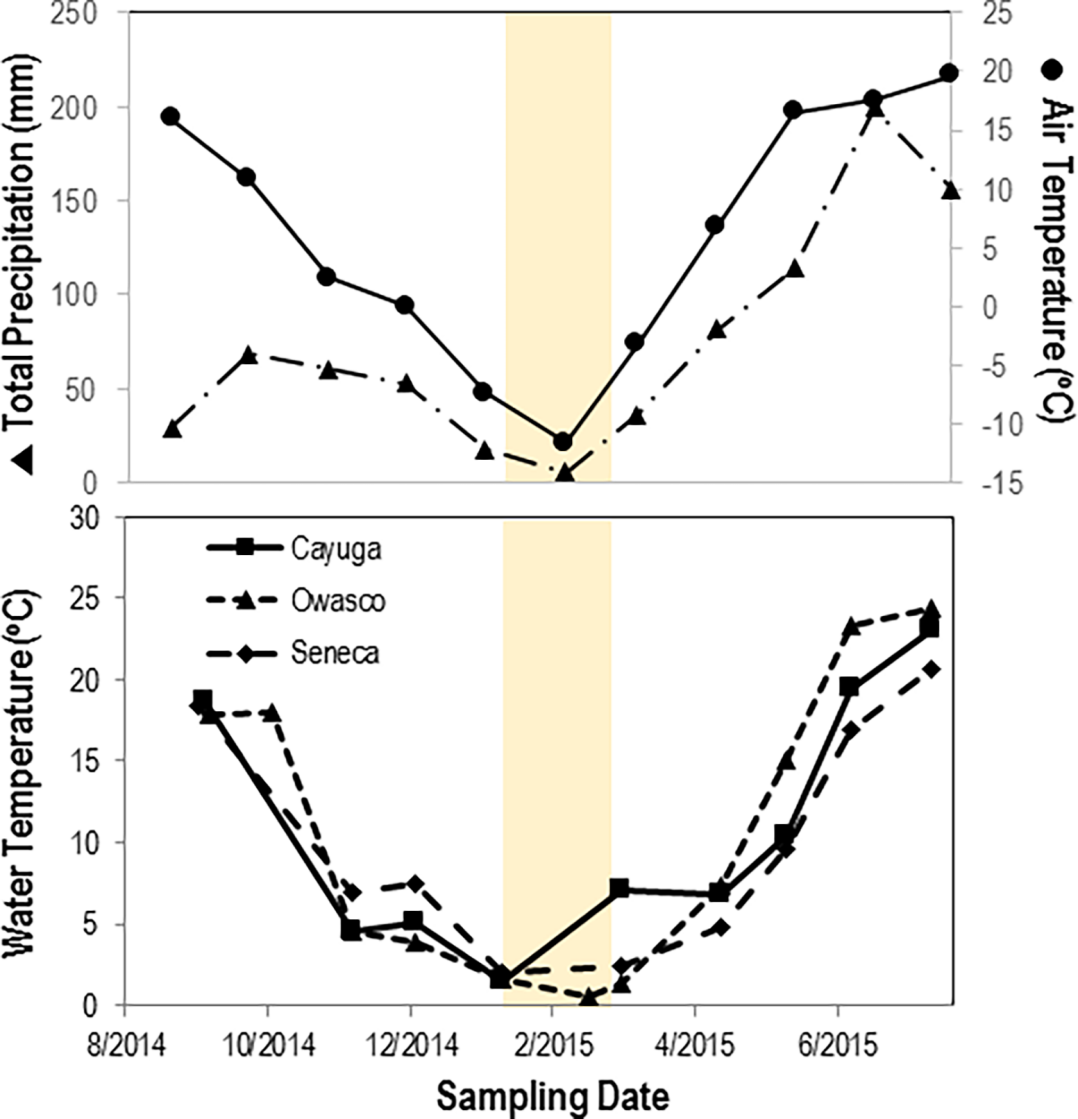
Physical and meteorological conditions at Cayuga, Owasco and Seneca Lakes over the sampling period. Meteorological data were taken from the National Weather Service station at the Ithaca Tompkins Regional Airport (https://www.ncdc.noaa.gov/). Temperature was determined by YSI handheld probe. The shaded region indicates approximate ice covered conditions.

All three lakes demonstrated a seasonal pattern of temperature consistent with mean air temperature (Fig 1). Lakes experienced ice cover for approximately 6 weeks from mid-December 2014 through mid-February 2015, followed by thaw in mid-February. The wettest month in the region occurred in mid-June 2015. The temperature at the sampling site in Cayuga Lake was higher in March 2015 than in other lakes, which may have been due to meltwater inputs from nearby creeks. Amongst the three lakes, Owasco had the greatest variation in water temperature from 24.4°C in mid-summer and 0.5°C in February, while Cayuga had the least variation from 23.1°C in mid-summer and 2°C in January. The overall change in temperature reflects polymictic conditions across all three lakes.

**Fig 2:**
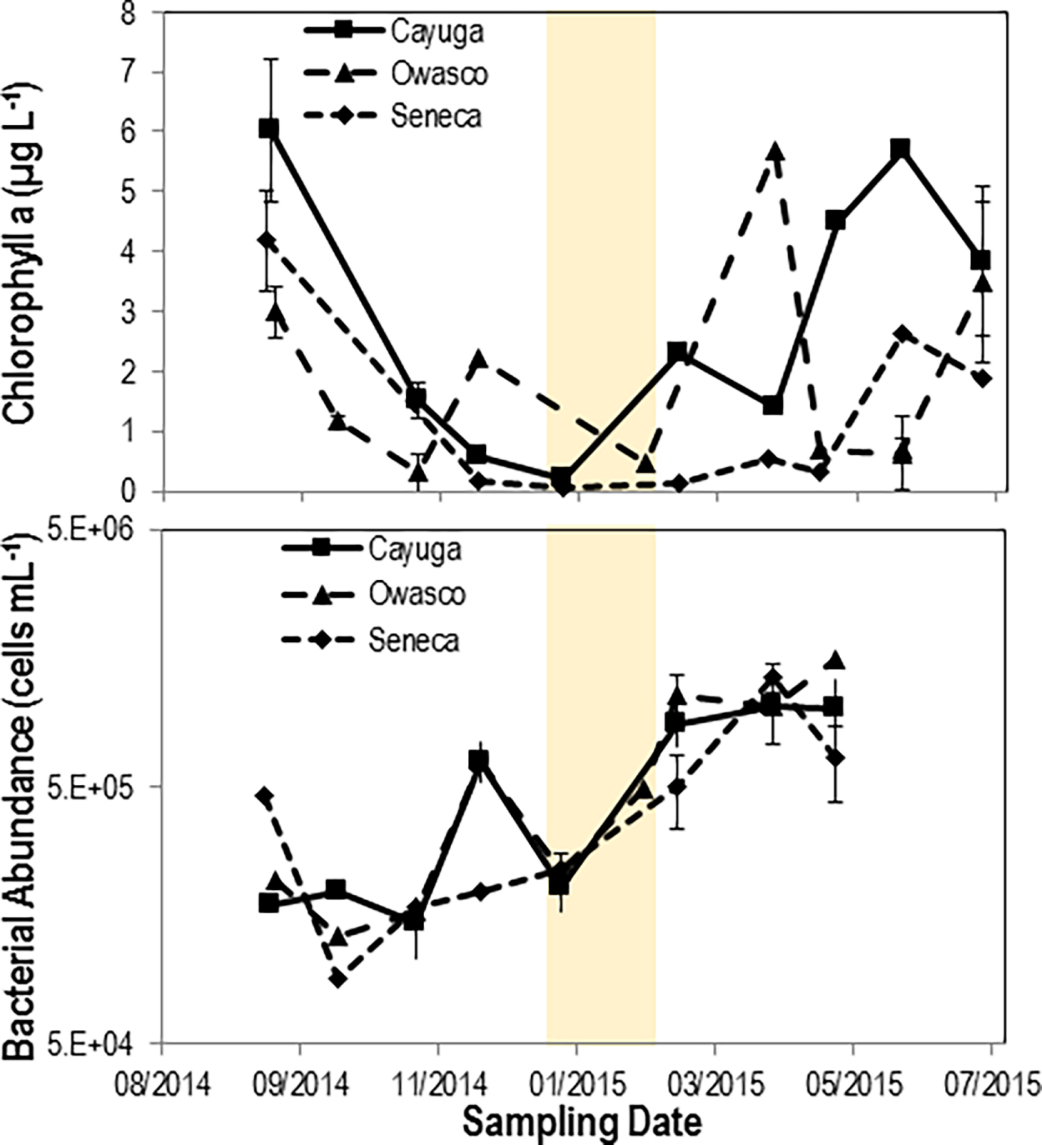
Chlorophyll a (top) and bacterial abundance (bottom) in Cayuga, Owasco and Seneca lakes during the sampling period. Chlorophyll a concentration was determined by acetone-extracted fluorometry. Bacterioplankton abundance was determined by DAPI staining and epifluorescence microscopy. The shaded region indicates approximate ice covered conditions.

Reductions in chlorophyll *a* concentration between August and December 2014 were concurrent with decreased water temperature across all three lakes. Elevated chlorophyll *a* from March to June 2015 suggested a spring bloom occurred (Fig 2). The timing of maximum phytoplankton concentration differed between lakes. Owasco experienced a spring bloom in April 2015, while both Seneca and Cayuga Lakes experienced blooms in May–June 2015. A pronounced ‘clear water phase’ after bloom exhaustion occurred in Owasco in May 2015. However, there was no clear water phase in Seneca and Cayuga over the sampling period. Bacterial abundance generally increased across all three lakes from Fall 2014 through Summer 2015. Bacterial abundance was greatest in Owasco Lake during the spring bloom in April 2015. Overall bacterial abundance did not demonstrate a clear seasonal pattern related to water temperature, with all three lakes maintaining high cell abundances during ice-covered months.

### Identification of RNA viral genotypes in plankton RNA libraries

An attempt was made to generate duplicate plankton RNA libraries from each lake monthly between September 2014 and May 2015. However, not all attempts were successful, due to poor nucleic acid recovery or sample preparation problems. A total of 30 environmental RNA libraries were successfully prepared, bearing a total of 9.13 x 10^7^ reads (S1 Table). Of these, viruses represented only a small (0.6%) fraction, with the remainder representing transcripts of cellular organisms (S1 Fig).

Global assembly of all 30 plankton RNA libraries generated 191,574 contigs that were ≥ 500 nt in length. Thirty-five contigs bore similarity to RNA viral genomes by tBLASTx against complete RNA genomes. Seventeen of these recruited ≥ 100 reads each in ≥ 2 libraries. Of these, 13 contigs produced significant alignments to eukaryote-associated RNA viruses by BLASTn against the non-redundant (nr) library at NCBI (Table 1; S2 Fig), while the remaining 4 produced significant alignments to bacteria or eukaryotes. The 13 viral contigs detected were predominately picornaviruses (n = 10), however 2 contigs matched most closely the putative tobamovirus associated with the green alga *Chara australis* [64] and one matched the Orungo virus (Reovirus) associated with mammals. Picornaviruses have a wide host range including microbial eukaryotes, plants, invertebrates and vertebrates. Tobamoviruses typically infect terrestrial plants. The genome of *Chara australis* virus bears similarity to both tobamoviruses and benyviruses, and infects a freshwater macrophyte [64]. These data demonstrate that viruses of metazoa may be common in freshwater ecosystems.

**Fig 3:**
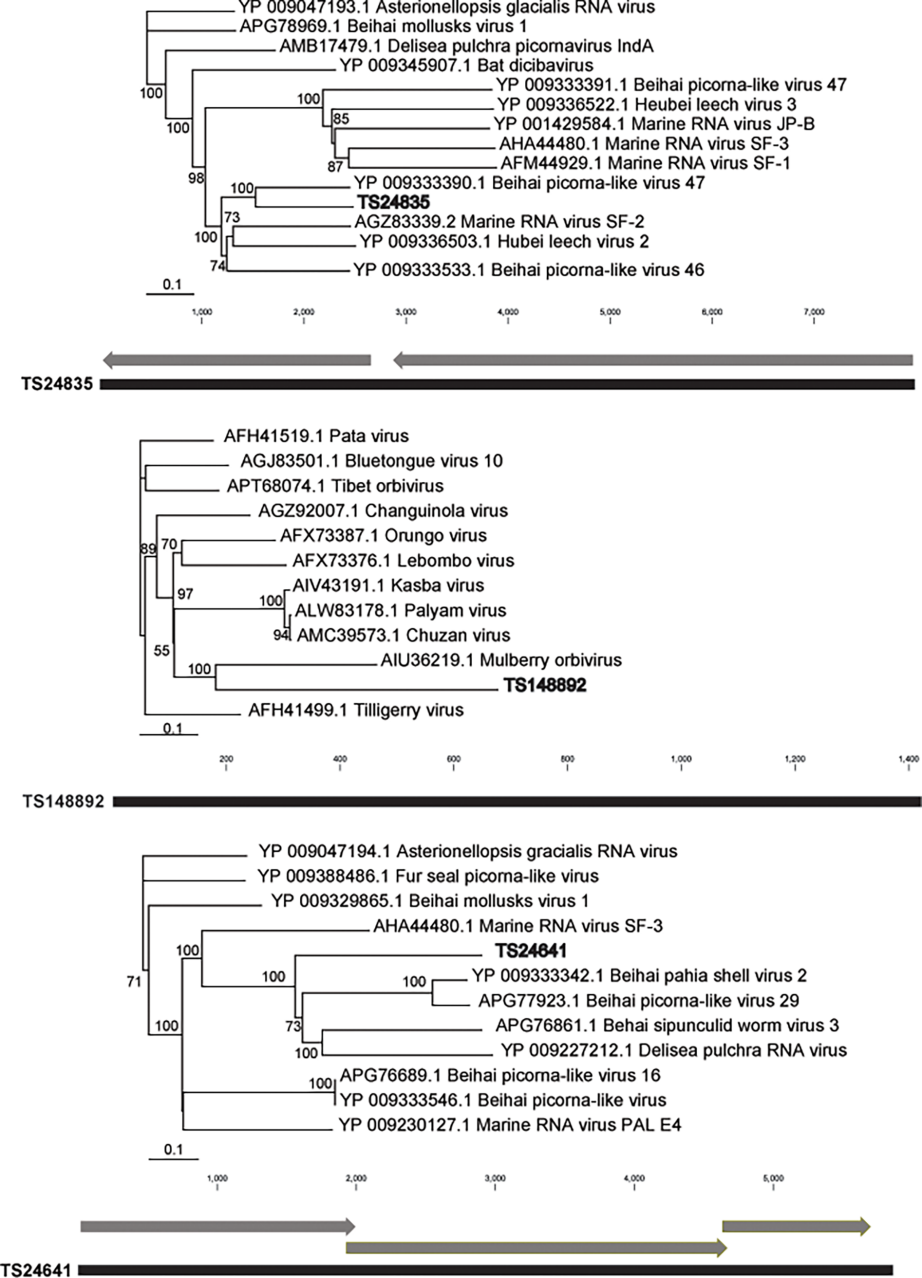
Contig architecture and pylogenetic representations of three viral genotypes observed in this study; Picornavirus TS24835 (top), Reovirus TS148892 (middle) and Picornavirus TS24641 (bottom). Phylogenetic dendrograms were generated based on an amino acid alignment (polyprotein for TS24835 and TS24635, and RNA polymerase VP1 for TS148892) using neighbor joining. Arrows indicate reading direction of ORFs on contigs.

**Fig 4:**
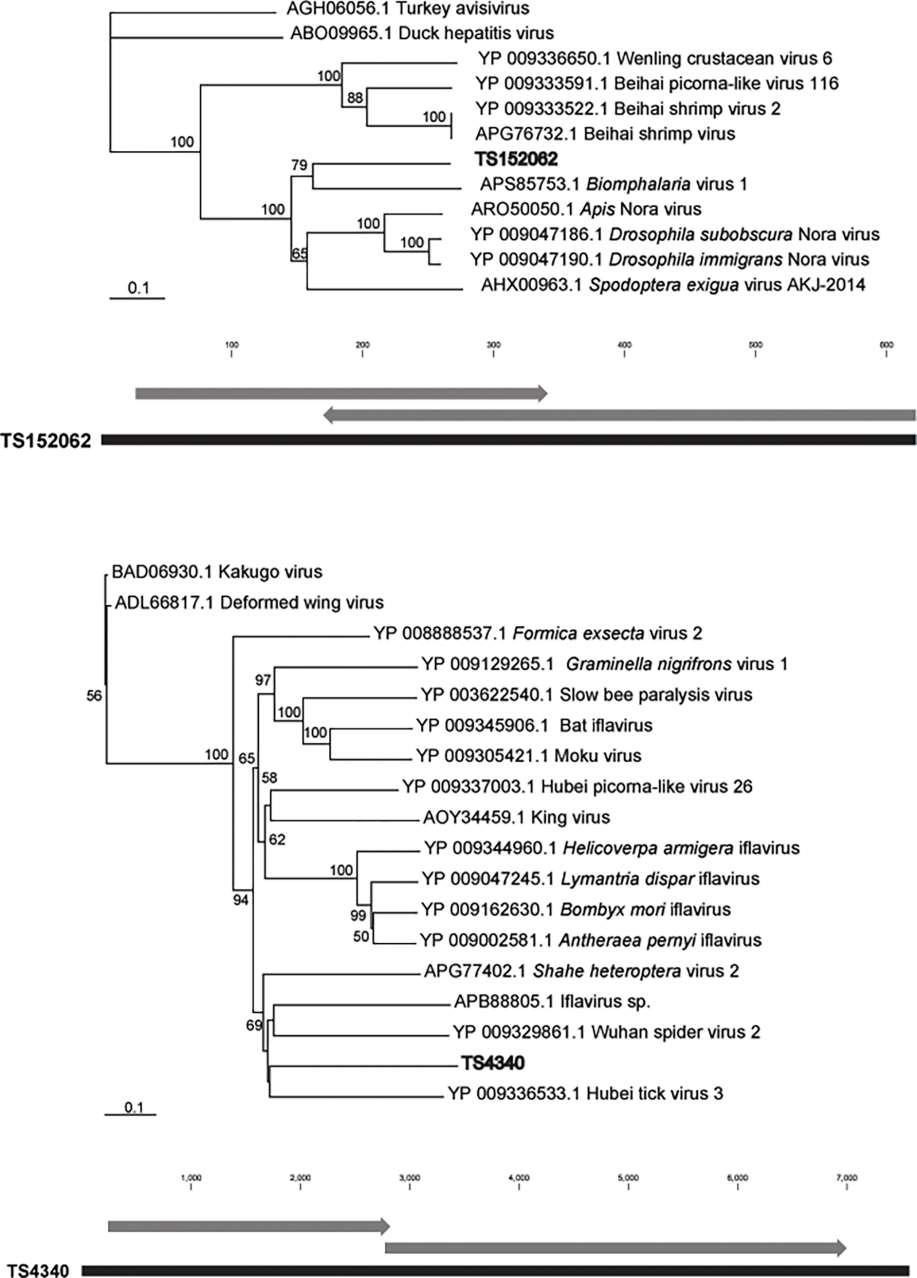
Contig architecture and phylogenetic representations of two viral genotypes observed in this study; TS152062 (top) and iflavirus TS4340 (bottom). Phylogenetic dendrograms (polyprotein) were generated based on an amino acid alignment using neighbor joining. Arrows indicate reading direction of ORFs on contigs.

We chose to focus on 5 viral genotypes (4 Picornaviruses and 1 Reovirus) that matched most closely metazoan RNA viruses at NCBI and were represented in ≥ 5 libraries. These were: 1) Contig TS152062, which was most similar to Wenling Crustacean Virus (APG78478.1, 44% amino acid ID, Picornaviridae) (Fig 3); 2) Contig TS4340, which was most similar to Bee Paralysis virus (YP_003622540.1, 24% amino acid ID, Iflaviridae) (Fig 3); 3) Contig TS24835, which was most similar to Behai picorna-virus, (APG78024.1, 44% amino acid ID, Picornavirales); 4) Contig TS148892, which was most similar to the Orungo Virus (AFX73387.1, 30% amino acid ID, Reoviridae); and 5) TS24641, which was most similar to Beihai Papia Shell Virus (APG78606.1, 45% amino acid ID, Picornavirales) (Fig 4). Phylogenetic analyses of these contigs indicated that all were most similar to metazoan-associated viruses, but were also homologous to viruses of unicellular eukaryotes (Figs 3 and 4). Picornaviruses are frequently encountered in aquatic plankton [65–67], and may represent pathogens of eukaryotic microorganisms or metazoans. They typically contain a small (~7–9 kBp) positive sense single stranded RNA genome, a non-enveloped capsid, and one or only a few non-overlapping open reading frames (ORFs, i.e. polyprotein gene organization). Four of the genotypes (TS24835, TS24641, TS152062 and TS4340) selected in this study belong to the order *Picornavirales*. TS24835 and TS24641 do not contain sufficient genome architecture or phylogenetic information to assign them to a specific family. TS4340 has genome architecture and amino acid sequence consistent with *Iflaviridae*. TS152062 is phylogenetically most similar to Nora viruses, which are within the *Picornaviridae*, but remains unclassified to family. Finally, TS148892 is most similar to orbiviruses (*Reoviridae*), which typically have segmented double stranded RNA genomes and represent arboviruses infecting vertebrates and invertebrates.

### Temporal quantification of RNA viral genotypes

**Fig 5:**
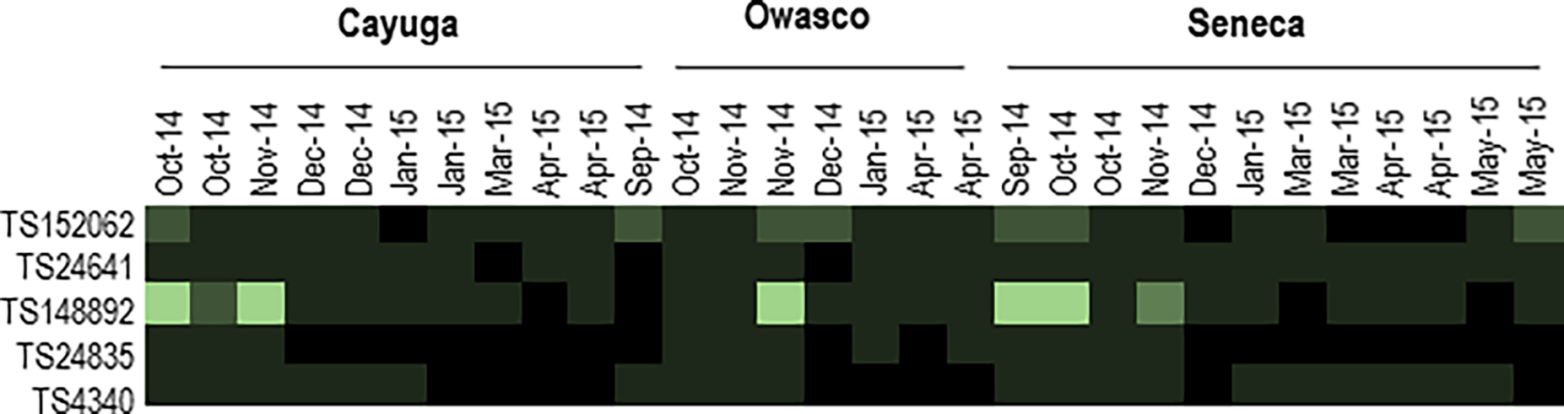
Heat map representation of reads recruiting from plankton RNA libraries against five candidate RNA viral genotypes. Read recruitment was performed in CLC Genomics Workbench 4.0 with minimum identity 0.95 and minimum overlap of 0.2. Brighter hues indicate a greater % of reads (standardized by column) than darker hues.

Read recruitment of all 5 viral genotypes revealed that reovirus TS148892 had the greatest representation in plankton RNA libraries (i.e. 5–0.2 μm) (Fig 5). All five contigs were better represented in plankton libraries during Fall 2014 than Winter or Spring 2015, with the exception of TS152062 in Seneca Lake (Fig 5). The representation of picornaviruses TS24841, TS24835 and iflavirus TS4340 in libraries was lower than the reovirus TS148892 and picornavirus TS152062 throughout the sampling period across all three lakes.

**Fig 6:**
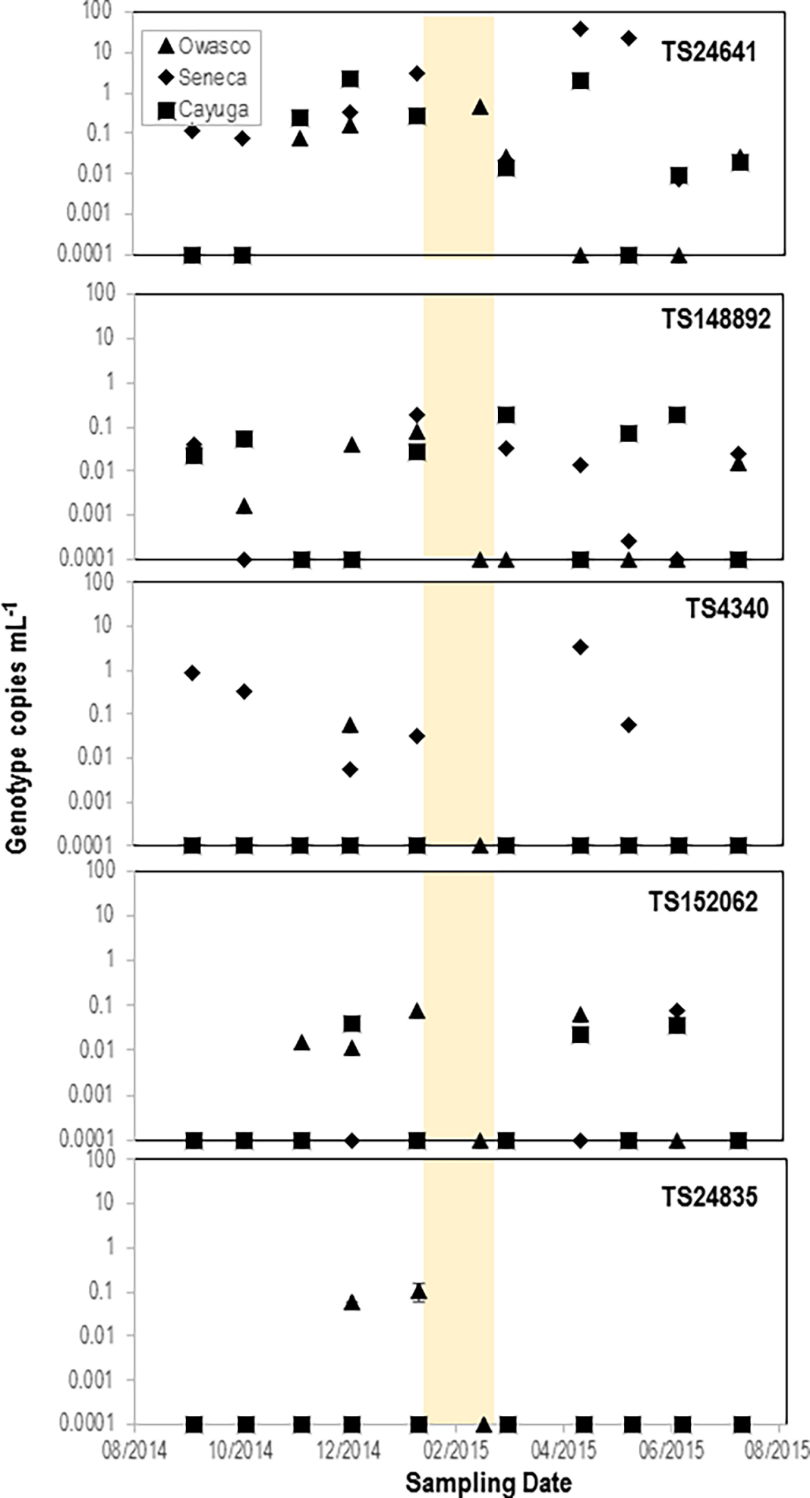
Abundance of 5 RNA viral genotypes in >5 μm size fraction plankton in Cayuga, Seneca and Owasco Lakes during the sampling period. Abundance of viral genotypes was determined by quantitative reverse transcriptase PCR (qRT-PCR). The practical detection threshold of all 5 primer/probe pairs was <0.001 genotype copies mL^-1^; abundances indicated at 0.0001 indicates that the genotype was not detected. The shaded region indicates approximate ice covered condition.

Quantitative PCR targeting the 5 genotypes was applied to the >5 μm size fraction cDNA from the time series samples (Fig 6). The five viral genotypes demonstrated different temporal patterns with season and between lakes. Picornavirus TS24641 had the greatest abundance during winter months in Owasco Lake, but had a very large abundance in Seneca and Cayuga during the spring (Mar-April 2015). Picornavirus TS24835 was absent entirely from Seneca and Cayuga Lakes but was abundant in winter months in Owasco Lake. Reovirus TS148892 was abundant in all 3 lakes throughout the sampling period. Picornavirus TS152062 was present after the onset of winter and into Spring in Owasco and Cayuga Lakes, however was absent from Seneca Lake. Iflavirus TS4340, on the other hand, had large abundances in Seneca Lake in both early Fall and Spring, but the genotype was only detected in late fall in Owasco Lake and was absent entirely from Cayuga Lake.

**Fig 7:**
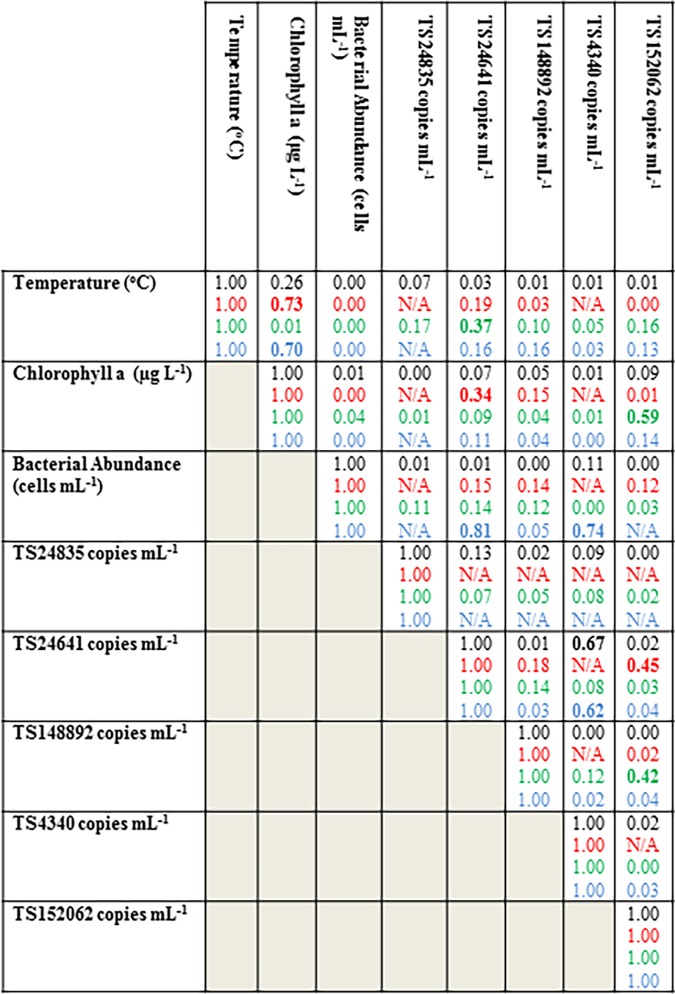
Correlation matrices between viral genotype abundance and environmental parameters in all lakes together; and in Cayuga Lake (red), Owasco Lake (green), and Seneca Lake (blue) separately. Numbers in bold represent significant correlations (Pearson’s ρ, p< 0.05).

These data provide evidence that the host of picornavirus TS24641 may be a large microscopic eukaryote or metazoan, since it was more abundant than other viral genotypes in the >5 μm size fraction than its representation in microbial size-fractioned plankton RNA libraries. Given that the virus was present year-round in all three lakes and demonstrated no temporal pattern with season, it is unlikely to infect phytoplankton, which exhibit decreased abundance during winter months. Furthermore, the lower abundance of this virus during spring in Owasco during a large bloom of phytoplankton, suggests it may target a heterotrophic organism (e.g. heterotrophic protozoa or metazoa). TS24641 abundance correlated with water temperature (R^2^ = 0.37) in Owasco Lake, with bacterial abundance (R^2^ = 0.81) in Seneca Lake, and weakly with chlorophyll *a* concentration (R^2^ = 0.34) in Cayuga Lake (Fig 7). These correlations reflect changes in overall productivity, suggesting that the host of TS24641 may respond to resource availability (e.g. nutrient conditions) and be controlled by overall temperature.

In contrast, picornavirus TS24835 was only detected in Owasco Lake in late fall and winter, which suggests that it may infect dying phytoplankton, or possibly a heterotrophic eukaryote consuming decaying phytoplankton. The higher relative contribution of TS24835 to microbial-sized plankton RNA libraries year-round, which contrasts with its pattern of abundance in the >5 μm size fraction, confirms its association with small a eukaryotic microorganism.

The seasonal abundance of reovirus TS148892 was greater than other viral genotypes in both the >5 μm size fraction throughout the year and microbial (5–0.2 μm) size fraction in the fall of 2014. Reoviruses infect a wide range of hosts, including fungi, plants, invertebrates and vertebrates. Since TS148892 occurred in both microbial and >5 μm size fractions and was especially enriched in fall samples, we speculate that it may infect phytoplankton or a eukaryote that consumes decaying primary production.

In contrast to reovirus TS148892, iflavirus TS4340 was only detected in two lakes (Owasco and Seneca), only during fall 2014 and spring 2015, and did not contribute substantially to microbial-sized libraries of plankton RNA (Fig 3). Our observation of TS4340 mainly in larger plankton is consistent with a metazoan host. The concordance of iflavirus TS4340 with elevated chlorophyll *a* in Seneca Lake in fall and spring suggests that it may infect a host influenced by enhanced resource availability. Zooplankton in the lake ecosystem exhibit seasonal abundance shifts according to resource availability, with peak abundances during spring and fall blooms, followed by declines in summer and fall [68, 69]. Iflavirus TS4340 correlated with Picornavirus TS24641 across all three lakes (R^2^ = 0.67). The correspondence between these viral genotypes may further indicate the presence of a potential pathogen of phytoplankton (TS24641) and a potential pathogen of its grazer (TS4340).

TS152062 was sporadically present in two lakes (Seneca and Owasco) and correlated with chlorophyll *a* concentration in Owasco lake (R^2^ = 0.59) but did not correlate with phytoplankton biomass in Seneca Lake. The absence of TS152062 from the >5 μm size fraction in early Fall 2014 and sporadic presence in Spring 2015 suggests that it may infect a rare host that was seldom captured in our survey. The larger contribution of this virus to microbial-size fraction plankton RNA libraries during months that it was absent in the >5 um size fraction by qPCR suggests that it either infects a smaller eukaryotic microorganism that form aggregates during enhanced phytoplankton production, or that it may be present in cell debris during peak phytoplankton abundance. TS152062 also correlated with picornavirus TS24641 in Cayuga Lake (R^2^ = 0.44), but did not significantly correlate with any genotype in Seneca Lake. A key question in our study was whether there is evidence for allocthonous viruses in lake plankton. Such viruses may be present on soil particles, animal carcasses or cell debris, or fecal matter that is washed into the lake by freshwater inputs or runoff [45, 70]. Of all viruses examined, picornavirus TS152062 had a temporal pattern consistent with enhanced rainfall or runoff. The abundance of picornavirus TS152062 was greatest after both rainfall peaks in September-October 2014 and after ice melt in March-April 2015. Given that picornavirus TS152062 was also abundant in the microbial-size fraction (5–0.2 μm) during rainfall events in early Fall 2014 and was present throughout the year at lower abundances, we hypothesize that TS152062 may infect an allochthonous host in nature. The similarity of TS152062 to a known virus of freshwater mollusk (*Biomphalaria* sp.) and several terrestrial arthropods (*Drosophila* sp., *Apis* sp. and *Spodoptera* sp.) is consistent with either a terrestrial or shallow-water invertebrate host.

Our data also provide evidence that some viruses were widespread between adjacent lakes, while others were specific to one or two lakes. For example, picornavirus TS24641 and reovirus TS148892 were present in all three lakes in >5 μm plankton, while iflavirus TS4340 and picornavirus TS152062 were present in larger plankton only two lakes, and picornavirus TS24835 detected in only one lake. All five viral genotypes were, however, observed in microbial size fraction (5–0.2 μm) libraries in all three lakes at least for some part of the sampling period. These data suggest viruses of larger unicellular eukaryotes or metazoans may persist in cell debris for some time after host lysis. Alternatively, our data could indicate that small eukaryotic hosts aggregate at certain times during the year and therefore are detectable in the larger size fraction. However, our data indicate that all viruses detected in our study are widely distributed across all three lakes.

## Conclusions

RNA viruses are constituents of all aquatic viral communities. However, their predicted hosts, seasonal dynamics and biogeography have not been extensively investigated in temperate lakes [50, 71]. Our data demonstrate that RNA viruses are widespread within regions, but may be temporally transient and associated with particles in different size fractions throughout the year. Most viruses observed in this study were likely associated with eukaryotic microorganisms, and we found evidence that these viruses may originate from metazoa within lakes or from the surrounding catchment. The presence of putative RNA viruses affiliated with eukaryotic microorganisms or metazoa piques interest into the role of viruses in host ecology, and more widely into their impact on freshwater ecosystem function.

## Supporting information

S1 Table Library and assembly characteristics for plankton RNA obtained from the 0.2–5 μm size fraction in three temperate lakes. (DOCX)

S2 Table Primers, hybridization probes and olignucleotide standards used to determine abundance of viral genotypes. (DOCX)

S1 Fig Percentage of viral reads amongst all reads annotated in community RNA libraries. Phylogenetic annotation occurred via the MG-RAST server ([72] as at May 2016) using an e-value cut-off of 0.001 (as at May 2016).
(TIF)

S2 Fig Heat map representation of total reads recruited to each of 13 contigs that matched most strongly RNA viruses at NCBI. Time Series library dates and locations can be found with reference to S1 Table.
(TIF)
