# Supplementary material for: Occurrence and seasonal dynamics of RNA viral genotypes in three contrasting temperate lakes
Source: PLoS One. 2018 Mar 15;13(3):e0194419. doi: 10.1371/journal.pone.0194419 (PMC5854377; doi:10.1371/journal.pone.0194419)
Supplement: S1 Table — (DOCX) [file pone.0194419.s001.docx]

**S1 Table :** Library and assembly characteristics for plankton RNA obtained from the 0.2 – 5 $\mu$m size fraction in three temperate lakes.

| **Sampling Date** | **Lake** | **Library Name** | **Total Reads** | **Assembled Reads** | **Contigs** | **Viral Reads** |
| --- | --- | --- | --- | --- | --- | --- |
| 10/16/2014 | Cayuga | TS6 | 1572388 | 957878 | 211 | 92 |
| 10/16/2014 | Cayuga | TS7 | 4268626 | 2326040 | 5034 | 4393 |
| 11/19/2014 | Cayuga | TS16 | 2521026 | 1898526 | 720 | 2176 |
| 12/15/2014 | Cayuga | TS17 | 2848306 | 1833968 | 5283 | 8520 |
| 12/15/2014 | Cayuga | TS19 | 3562064 | 1673965 | 4519 | 3316 |
| 1/21/2015 | Cayuga | TS22 | 2612812 | 1084950 | 2430 | 111 |
| 1/21/2015 | Cayuga | TS26 | 4318510 | 2531163 | 8039 | 6029 |
| 3/12/2015 | Cayuga | TS42 | 1099760 | 473239 | 324 | 31 |
| 4/23/2015 | Cayuga | TS32 | 2112764 | 834726 | 292 | 0 |
| 4/23/2015 | Cayuga | TS36 | 1338228 | 571035 | 421 | 17 |
| 9/15/2014 | Owasco | TS2 | 1783468 | 1258778 | 456 | 38 |
| 10/16/2014 | Owasco | TS12 | 3964910 | 2654771 | 2588 | 5628 |
| 11/19/2014 | Owasco | TS14 | 3446962 | 2424229 | 1607 | 2042 |
| 11/19/2014 | Owasco | TS9 | 2628222 | 1657361 | 1287 | 1254 |
| 12/15/2014 | Owasco | TS20 | 2262568 | 1866390 | 642 | 5139 |
| 1/21/2015 | Owasco | TS24 | 4946644 | 2458515 | 5497 | 23896 |
| 4/23/2015 | Owasco | TS28 | 2328598 | 1468173 | 578 | 5 |
| 4/23/2015 | Owasco | TS34 | 3744518 | 1865575 | 4842 | 1078 |
| 9/15/2014 | Seneca | TS3 | 5551078 | 3981638 | 1076 | 28 |
| 10/16/2014 | Seneca | TS4 | 4123670 | 2025012 | 547 | 156 |
| 10/16/2014 | Seneca | TS5 | 4638522 | 3253969 | 2953 | 856 |
| 11/19/2014 | Seneca | TS10 | 5108582 | 3800247 | 2407 | 1076 |
| 12/15/2014 | Seneca | TS23 | 3155836 | 1719001 | 3778 | 4203 |
| 1/21/2015 | Seneca | TS27 | 4246086 | 2356319 | 5211 | 982 |
| 3/12/2015 | Seneca | TS21 | 3240522 | 1687218 | 1427 | 7 |
| 3/12/2015 | Seneca | TS25 | 2993190 | 1749169 | 3841 | 383 |
| 4/23/2015 | Seneca | TS39 | 2256742 | 1156685 | 12339 | 5378 |
| 4/23/2015 | Seneca | TS40 | 1844156 | 980668 | 10123 | 6001 |
| 5/20/2015 | Seneca | TS45 | 1587240 | 676573 | 655 | 122 |
| 5/20/2015 | Seneca | TS46 | 2032384 | 1344188 | 310 | 699 |
|  |  | Total | 92138382 | 54569969 | 89437 | 83656 |
|  |  |  |  |  |  |  |
