# Supplementary material for: Occurrence and seasonal dynamics of RNA viral genotypes in three contrasting temperate lakes
Source: PLoS One. 2018 Mar 15;13(3):e0194419. doi: 10.1371/journal.pone.0194419 (PMC5854377; doi:10.1371/journal.pone.0194419)
Supplement: S2 Table — (DOCX) [file pone.0194419.s002.docx]

**S2 Table :** Primers, hybridization probes and olignucleotide standards used to determine abundance of viral genotypes.

| **Viral Genotype** | **Forward Primer (5’ – 3’)** | **Reverse Primer (5’ – 3’)** | **Probe (5’ – 3’)** | **Oligonucleotide Standard (5’ – 3’)** |
| --- | --- | --- | --- | --- |
| TS24641 | CGTTACGCCTTTCAGGTTTT | ACTTCAGCTGCCAAACCACT | GCCATTAATTCGGGTTATACGCATGA | TTTATACAGAACAATTGGTTGGCTTGTGAGTGCGCAGTAGGAATCAACGCGCACGGAAGACAGTGGAATCAATTAGCTACTCACATGCTCGAACATCCTAATTATATAGCCGGCGATTATAAAGCTTATGACAAGCAGGTTGGACCAAAAGTTATGCGTTACGCCTTTCAGGTTTTAATTGACGTAGCCATTAATTCGGGTTATACGCATGAAGATGTTACGATTATGAGTGGTTTGGCAGCTGAAGTTTGTTTTCCATTATATGAATATGATGGTGTTTTTATACAATTATTTGGCTCCAATCCCTCCGGACACCCTTTGACGGTTATATTAAATAATTTAATCAATAGCATTTATATGAGATATGTTTATAGTTTTAATTATGACATATCAACTTTTTCCAACAACGTTAATATTGTGTGCTATGGTGACGACAATATTTTATCAGTCAGTAATAAAGTTCCCGATTTCAACTTTAATACTATACGAATAACTTTAGCT |
| TS24835 | ACTGTCAACCACACGTACCG | GTTTTGGGGATCCAATCCTT | CAGGGAGTGAGCAATGCAGTTAATGA | ATTTTTGATATGGATTTTTCATTTAATGCTGCCATGTGACTTCCAATTTCCTCATCATAACGCCATGCCCGTTGGAGAAACACGATGTCATTCATATTTAGGAAAGGTACAGAGTTACTCTCCTTATCTGCCATAGTGTATTTAACACCAATTTTACCCAATTCCCTCTGGATAACAGTGTGGTCATAAGTAGTAATTTCAGCACTGACGTTTAATACATCATCATCACCATAGGTGATGAGAGCAACGTAAGTTTTAAAATCGCCAAGAATGAGACCCACTGTCAACCACACGTACCGCATATACAGGGAGTGAGCAATGCAGTTAATGATGACAGTCAGAGGATGTCCTGAAGGATTGGATCCCCAAAACTCGACCAAATCTCCGTTGAAATCACACAGGGGAAAAGCTACATCTTCAGCTAAACCTAGGATAACCATAATATCTGCATTTGGCCATCCAGCATGCTTCAAAATTTTGATGATGAATTGAAAGGCTGCTAAAATCCAGACTCCTGACATACGTTTATCATATTTGGAGAAATCACCAGCAATGTTTCGATTAATACCAAACTGTGTGAGATAATGGTACAATTCATCCCATTCGTAAGATGTAGTATTTGTACCAGGTGCAGCTTCAAAAATGAATTTATTATTCTGAACTACACGAACAAATGAGAGCAAGT |
| TS4340 | GCAGGAACGGAATATTGGAA | TTTTGTTGTCGGTGAACTGC | CAACTATTTTGCACAAGACGCGGTT | TGTATCTGGGAGGAGAACCGGGAGTCGGGAAATCTTTCGTGCTCGACCCGATAATATCAGCAGTACTTCAAGAAGCAGGGGTCGAGAACGCAGTGTCAAACATCTGGACAAGGAACGCAGGAACGGAATATTGGAACAACTATTTTGCACAAGACGCGGTTAAATATGATGACTTTGGCGCAGTTCACCGACAACAAAACTCTGAAGGAGCAGAGCTTATTGCACTGAAGACTAGCGCAGTATTTAACCCACCTTTTGCAGCAATTCCAGACAAGAACAGACTCGCAAATCCAAGGATCGTGGCAATCGCATCTAATTCACTT |
| TS152062 | TTGCTTTCGATGTCTGCTTG | ATGACTCCGATCACCCTCAG | CGCTTGACGAGATTTTGGACAAAAA | TTGCTTGGTATGGTCGGCCTTTGCTTTCGATGTCTGCTTGAACAGTTAGTACTACGCTTGACGAGATTTTGGACAAAAACTGAGGGTGATCGGAGTCATCGGAGCGGCTAAATATGTCGTCTACATAATGCACTTCCTGTCCTATGTATCCCTGGTCGTAATTGTCATCTGCGTTTTGGGACCACATCGTCCAATTCTGCACGTCGTCCAGTAGAGGCTGTAAAGCTACATTGGTTGCGGCACGCGTTGCTAAGCGTTGGCGGATGAGTTGTATGAGGAAGGTGCTCAGGAGAGACTTTCCT |
| TS148892 | TCCTCGAGAGTGTTTTTCCA | CGGAGAATGGGAAATTTATTGA | TCATCAACGACGCATAGCTCATCA | ATTAGAAATACAACAGCCGACCCCCCCATGTGCAGACGCGCAATGAGTCACAATGGGTGAGATCAACATATGTATTCTAGTTGCTAAGTCGTTACTATCATCATATATTATTTTATCATTCAACATACGCGATCGCCCTATTCTGGTGGATGCCAGAGTTATAGCCATCGTATACATTTGCAATAATCCTAAATCAAATTTCAATCCTCGAGAGTGTTTTTCCATCATCAACGACGCATAGCTCATCAAAAAAGCTTGTATATCATCAATAAATTTCCCATTCTCCGACGAATGAATCATGATTCTGTCTTGTGGAATAAGAATCCCACATTTAGCATGAGTCTGTCTGAA |
