# Supplementary figures and images for: Occurrence and seasonal dynamics of RNA viral genotypes in three contrasting temperate lakes
Source: PLoS One. 2018 Mar 15;13(3):e0194419. doi: 10.1371/journal.pone.0194419 (PMC5854377; doi:10.1371/journal.pone.0194419)

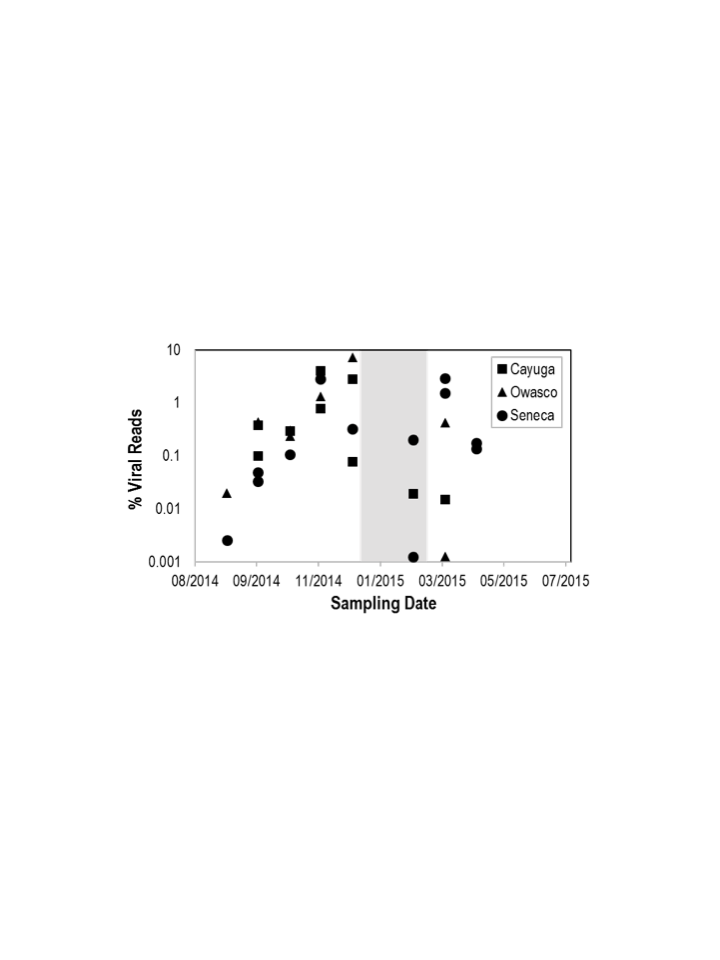

Supplement: S1 Fig — Phylogenetic annotation occurred via the MG-RAST server ([72] as at May 2016) using an e-value cut-off of 0.001 (as at May 2016). (TIF) [file pone.0194419.s003.tif]

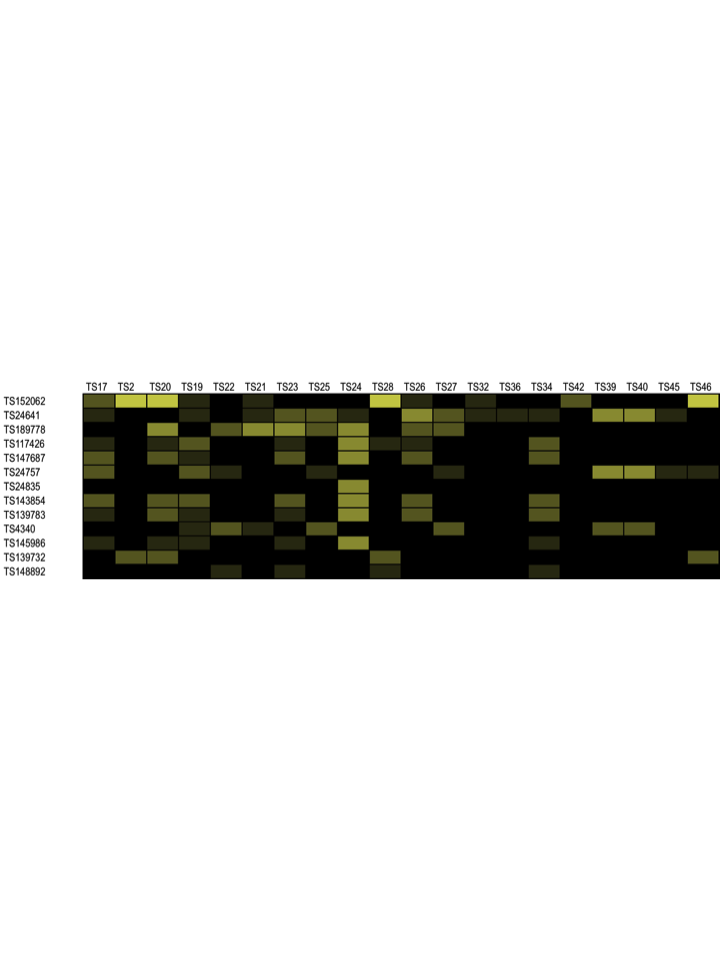

Supplement: S2 Fig — Time Series library dates and locations can be found with reference to S1 Table. (TIF) [file pone.0194419.s004.tif]
